# Association between cardiac autonomic dysfunction, cognitive impairment, and survival in patients with amyotrophic lateral sclerosis

**DOI:** 10.1007/s10286-025-01112-0

**Published:** 2025-03-08

**Authors:** Zehui Li, Jingjing Fan, Zhenxiang Gong, Jiahui Tang, Yuan Yang, Mao Liu, Min Zhang

**Affiliations:** 1 Department of Neurology, Tongji Hospital, Tongji Medical College, Huazhong University of Science and Technology, Wuhan, 430030 Hubei China; 2 Department of Cardiology, Tongji Hospital, Tongji Medical College, Huazhong University of Science and Technology, Wuhan, 430030 Hubei China; 3 Department of Neurology, The First Affiliated Hospital of Xiamen University, Xiamen, China; 4 Department of Neurology, The Central Hospital of Enshi Tujia and Miao Autonomous Prefecture, Enshi, China; 5 Department of Neurology, SUNY Downstate Health Sciences University, Brooklyn, NY USA; 6 Department of Neurology, Shanxi Bethune Hospital, Shanxi Academy of Medical Sciences, Third Hospital of Shanxi Medical University, Tongji Shanxi Hospital, Taiyuan, 030032 Shanxi China; 7 Hubei Key Laboratory of Neural Injury and Functional Reconstruction, Huazhong University of Science and Technology, Wuhan, 430030, Hubei, China

**Keywords:** Amyotrophic lateral sclerosis, Cognitive impairment, Cardiac autonomic dysfunction, Heart rate variability

## Abstract

**Purpose:**

The aim of this study was to investigate the relationship between cardiac autonomic dysfunction, cognitive impairment, and survival in patients with amyotrophic lateral sclerosis (ALS).

**Methods:**

The heart activity of 65 patients with ALS (28 with normal cognition [ALS-CN]; 37 with impaired cognition [ALS-CI]) and 38 healthy controls (HCs) was measured by 24-h Holter monitoring. Heart rate (HR) measures and heart rate variability (HRV) parameters were compared between the three study groups and, additionally, correlated with five Edinburgh Cognitive and Behavioral ALS Screen (ECAS) domains in the ALS subgroups. Age, gender, and educational level were adjusted. Factors associated with cognitive status were assessed using logistic regression. Survival predictors in patients with ALS were analyzed using the Kaplan–Meier estimator and Cox regression.

**Results:**

Compared to the HCs, patients with ALS-CI exhibited lower RRI (R-R-interval; *P* = 0.017), SDNN (standard deviation of all normal RR intervals; *P* = 0.013), SDNN Index (*P* = 0.044), and VLF power (very low-frequency power; *P* = 0.012). Total power was reduced in the ALS-CI group compared to the HCs (*P* = 0.036) and ALS-CN group (*P* = 0.048). In patients with ALS-CN, language negatively correlated with mean HR (*P* = 0.001) and positively with the RRI (*P* = 0.003), SDNN (*P* = 0.001), SDANN (standard deviation of the average NN intervals; *P* = 0.005), total power (*P* = 0.006), VLF power (*P* = 0.011), and low-frequency power (*P* = 0.026). Visuospatial function correlated positively with the SDNN Index (*P* = 0.041). In patients with ALS-CI, executive function (*P* = 0.015) and ECAS total score (*P* = 0.009) negatively correlated with the RMSSD (square root of mean sum-of-squares of differences between adjacent NN intervals), while visuospatial function correlated positively with normalized LF value (LFnu; *P* = 0.049). No associations were observed between the other cognitive domains and any of the 14 HRV/HR measures in patients with either ALS-CI or ALS-CN*.* SDNN ≤ 100 ms was linked to cognitive impairment (*P* = 0.039) and also showed a borderline association (*P* = 0.066) with poorer survival, while cognitive impairment (*P* = 0.010) was significantly linked to worse outcomes.

**Conclusions:**

Patients with ALS with cognitive impairment demonstrated reduced cardiac autonomic modulations and altered cognitive autonomic associations. Cognitive impairment was linked to reduced survival, with baseline SDNN ≤ 100 ms identified as a potential marker.

**Supplementary Information:**

The online version contains supplementary material available at 10.1007/s10286-025-01112-0.

## Introduction

Amyotrophic lateral sclerosis (ALS) is a neurodegenerative disease characterized by a poor prognosis and an average survival time of 3–4 years [1]. This disease has long been known to affect upper and lower motor neurons, leading to muscle weakness and atrophy, but recent studies have revealed the involvement of other systems as well [2]. Approximately 10–15% of patients with ALS also meet the diagnostic criteria for frontotemporal dementia (FTD) [3], giving rise to the degenerative disorder referred to as amyotrophic lateral sclerosis frontotemporal spectrum disorder (ALS-FTSD) [4]. Cognitive dysfunction significantly impacts the quality of life of ALS patients and their caregivers [3].

The Edinburgh Cognitive and Behavioral ALS Screen (ECAS) is an assessment tool encompassing various cognitive subdomains that is employed to evaluate cognitive dysfunction in ALS patients of different ethnicities [5–9]. However, no biomarkers have been incorporated into clinical practice for assessing cognitive dysfunction in ALS patient populations [10, 11]. Education level and age are known to be pivotal factors in determining cognitive status [10]. Additionally, cognitive impairment in ALS patients has been associated with decreased levels of uric acid [6], diminished counts of peripheral blood CD4^+^ T lymphocytes, CD8^+^ T lymphocytes, and B lymphocyte counts [12], as well as altered profiles of the gut microbiome and bile acid levels [13].

Previous studies have demonstrated that cardiac autonomic function is linked to cognitive performance not only in healthy populations [14] but also among individuals with traumatic brain injury (TBI) [15, 16], dementia [17], and chronic fatigue syndrome (CFS) [18]. Various studies have also found cardiac autonomic dysfunction in ALS patients [19–24]. Additionally, one recent study showed that urinary complaints could predict poor survival in ALS patients [25], and two previous studies suggested that behavioral abnormalities could be related to increased mortality [26, 27]. It is currently unclear whether cardiac autonomic dysfunction plays a role in cognitive impairment in ALS patients and whether the coexistence of cardiac autonomic dysfunction and cognitive impairment might influence long-term survival. Such knowledge is particularly important as cardiac autonomic dysfunction has been shown to be associated with an increased risk of cardiac events and a worse clinical prognosis in various pathologies [28–32].

The aim of the present study was to investigate the relationship between cardiac autonomic function and cognitive function and to evaluate whether baseline cardiac autonomic dysfunction and cognitive impairment could help predict survival in ALS patients.

## Methods

### Subjects and clinical data

From November 2019 to September 2022, 65 patients with clinically possible, probable, and definite ALS, respectively, based on the EI Escorial criteria for the diagnosis of ALS [33], and 38 healthy controls (HCs) were enrolled in the study. Follow-up was conducted via telephone and outpatient visits. Death was used as the study endpoint. Exclusion criteria included a family history of ALS with one first-degree relative or two or more other relatives diagnosed with ALS; a subsequent diagnosis of FTD; history of diseases that could potentially cause autonomic dysfunction, including cardiac and cerebrovascular diseases (coronary artery disease, myocardial infarction, atrial fibrillation, atrioventricular block, hypertension, stroke, transient ischemia attack), diabetes, insomnia, hyperthyroidism, and traumatic brain injury; extrasystole rate of > 5% for the entire Holter recording; history of cognitive impairment due to other diseases; and another neurological diagnosis. HCs were randomly selected from family members of patients undergoing 24-h Holter monitoring; exclusion criteria for the HCs were similar to those for ALS patients. Clinical data, including name, gender, age, body mass index [34], education level, past medical history, medication use, social history, site of onset, disease course (from disease onset to study enrollment), King’s Clinical Staging, and depressive symptoms (Beck’s Depression Inventory, with score ≤ 10 defined as negative and score > 10 defined as positive) [35], were collected. The assessment of disease severity was conducted using the Amyotrophic Lateral Sclerosis Functional Rating Scale (ALSFRS-R)-Revised (ALSFRS-R) [36]. None of the patients were bedridden at study enrollment.

This study was approved by the Ethics Committee of Tongji Hospital, Tongji Medical College, Huazhong University of Science and Technology (TJ-IRB20201222) and adheres to the principles outlined in the Declaration of Helsinki [37]. Informed consent was obtained from either the patients or their legal representatives.

### Cognitive assessment

All patients diagnosed with ALS and 34 of the 38 HCs (4 healthy participants refused) underwent cognitive assessment using ECAS [10]. The evaluations were conducted in a controlled environment with warm lighting and minimal noise disturbance. The ECAS comprises both ALS-specific and ALS-nonspecific domains to evaluate various cognitive functions. Specifically, the ALS-specific domains encompass language skills (naming, understanding, spelling), verbal fluency, and executive function (reverse digit span, alternation, sentence completion, social cognition), while the ALS-nonspecific domains include memory (immediate recall, delayed recall, delayed recognition) and visuospatial function (dot counting, cube counting, number location). A higher score on the ECAS scale indicates higher cognitive performance. Based on the ECAS cutoff total score of 81.92 points proposed by Ye et al. [10], patients with ALS participating in the present study were further categorized into a subgroup with normal cognition (ALS-CN; ECAS total score ≥ 81.92 points) and a subgroup with cognitive impairment (ALS-CI; ECAS total score < 81.92 points) [10]. The same cut-off score was used to identify HCs with underlying cognitive impairment, although none of the HCs in the present study had subjective cognitive complaints. Of the 34 HCs who undertook cognitive testing using ECAS, four had total score < 81.92 points and were thus excluded from further statistical analysis.

### Heart rate variability

The 24-h Holter monitoring was conducted using an ambulatory device (DMS300-4AL; DM Software, Tustin, CA, USA) at a sampling rate of 4096 Hz. Participants were able to engage in daily activities while wearing this device. None of the participants reported significant episodes of nocturnal insomnia during the recording period. Raw data were extracted from the Holter recordings and imported into DMS software for further analysis. Electrocardiogram (ECG) signals were visually inspected by a trained technician, who manually removed any artifacts using the DMS software. The processing of ectopic heartbeats involved three steps: (1) identification of ectopic RR interval(s); (2) identification of two RR intervals preceding and following the ectopic heartbeat/s; and (3) exclusion of the identified segment entirely from the heart rate variability (HRV) analysis.

RR interval (RRI) was calculated as the reciprocal value of heart rate (HR). Time-domain parameters included standard deviation of all normal-to-normal (NN) intervals (SDNN), standard deviation of average NN intervals in all 5-min segments (SDANN), mean standard deviations of all NN intervals for each 5-min segment in a 24-h HRV recording (SDNN Index), square root of mean sum-of-squares of differences between adjacent NN intervals (RMSSD), and a number of pairs with adjacent NN intervals differing by > 50 ms divided by the total number of all NN intervals (pNN50). Frequency domain parameters included total power (TP, in ms^2^), very low-frequency power (VLF power, in ms^2^; 0.003–0.04 Hz), low-frequency power (LF power, in ms^2^; 0.04–0.15 Hz) and its normalized value (LFnu: LF power/(LF power + HF power) × 100%), high-frequency power (HF power, in ms^2^; 0.15–0.40 Hz) and its normalized unit (HFnu: HF power/(LF power + HF power) × 100%), and LF/HF (ratio of LF power to HF power). A cut-off SDNN value of 100 ms was used to categorize patients into groups with reduced SDNN (≤ 100 ms) or normal SDNN (> 100 ms) [30, 31, 38–41]; no patients had SDNN ≤ 50 ms. Electronic Supplementary Material (ESM) Table S1 provides a comprehensive overview of individual HRV parameters and their physiological implications.

The HRV analysis was conducted on the ECG dataset for the entire 24-h monitoring period, as well as separately for daytime and nighttime periods.

### Statistical analysis

The Shapiro–Wilk test was utilized to evaluate the data distribution of all parameters. For three-group comparisons, normalized data were analyzed using analysis of variance (ANOVA), while non-normalized data were assessed using the Kruskal–Wallis test. Post-hoc analysis was conducted using the Bonferroni test with adjusted* P* values. HRV data were additionally compared between the three groups (with Blom transformation applied for non-normally distributed data) using analysis of covariance (ANCOVA) to control for confounding factors, including age, gender, and educational level. For two-group comparisons, the unpaired Student’s t-test was used for normalized data, and the Mann–Whitney U-test was used for non-normalized data. Fisher’s test was used for categorical variables. Partial correlation was performed to examine the relationship between ECAS total or subdomain scores and HRV parameters by controlling for covariates, including age, gender, and educational level. Multivariate logistic regression was used to investigate the associations of demographic factors and SDNN grouping with cognitive impairment in ALS patients. Kaplan–Meier estimator and multi-variable Cox regression analysis were used to evaluate whether cognitive status and SDNN grouping could affect survival in ALS patients. Normally distributed data are presented as the mean ± standard deviation (SD), while non-normally distributed data are presented as the median with minimum–maximum values [min—max] [28, 29]. Statistical analyses were conducted using SPSS statistical software version 25.0 (SPSS IBM Corp., Armonk, NY, USA), with the significance level set at *P* value < 0.05. GraphPad version 8.3.0 software (GraphPad Software, San Diego, CA, USA) was used to create survival curves.

## Results

### Demographic characteristics

The median disease duration among ALS patients was 10 months, and the ALSFRS-R score was 41. There were no differences in BMI, disease duration, ALSFRS-R score, bulbar or respiratory involvement, King’s clinical staging, or impaired fasting glucose between the two ALS subgroups (Table 1). No differences were observed in age, gender, depressive symptoms, and tobacco or alcohol use between the three groups (Table 1). Patients in the ALS-CI subgroup had lower levels of education (median [min–max] 7 [0–12] years) compared to those in the ALS-CN subgroup (median 9 [5–16] years; *P* = 0.001) and HCs (median 9 [6–19] years; *P* < 0.001; ESM Table S2). ECAS total and subdomain scores were consistently lower in ALS-CI patients than in ALS-CN patients, as shown in ESM Table S6. Additional detailed demographic data are presented in Table 1. Of the 65 ALS patients, follow-up data were available for 56 patients, with nine patients lost during follow-up; for the remaining 47 patients, follow-up data were available up to February 2024 (median duration ALS: 22.5 months).

**Table 1:** Demographic data for patients with amyotrophic lateral sclerosis and healthy controls

Demographic data	Study (sub)groups			*P* value
	HCs (*n* = 30)	ALS-CN (*n* = 28)	ALS-CI (*n* = 37)^a^	
Age (year)	52.87 ± 8.81	53.00 ± 10.467	54.00 ± 7.808	0.291
Male (%)	20 (66.7)	19 (67.9)	17 (45.9)	0.141
Education (year)	9 [6–19]	9 [5–16]	7 [0–12]^&,#^	< 0.001*
Duration (month)	–	10 [2–48]	10 [1–57]	0.661
ALSFRS-R	–	42 [26–48]	41 [23–47]	0.894
Bulbar involvement (%)	–	16 (57.1)	20 (54.1)	0.804
King’s Clinical Staging (Stage 1/2/3/4)	–	7/11/8/2	5/23/7/2	0.305
Respiratory involvement (%)	–	3 (10.7)	2 (5.4)	0.426
Depressive symptom (%)	7 (23.2)	5 (17.9)	13 (35.1)	0.283
*BMI (kg/m*^*2*^)				
< 25	−	23 (82.1%)	28 (75.7%)	0.530
≥ 25	–	5 (17.9%)	9 (24.3%)	
*Past medical history (%)*				
Hyperlipidemia	0	1 (3.6)	0	/
Impaired fasting glucose	0	1 (3.6)	3 (8.1)	0.628
*Medication use (%*)				
Antihyperlipidemic agents	0	1 (3.6)	0	/
*Social history (%)*				
Tobacco use	7 (23.3)	7 (25.0)	6 (16.2)	0.662
Alcohol use	2 (6.7)	3 (10.7)	1 (2.7)	0.372

Values in table are presented as the mean ± standard deviation, number with the percentage in parentheses, and the median with minimum–maximum values in square brackets, as appropriate

* ALS* Amyotrophic lateral sclerosis, *ALS-CI* subgroup of ALS patients with impaired cognition, *ALS-CN* subgroup of ALS patients with normal cognition, *ALSFRS-R* ALS Functional Rating Scale-Revised,* BMI* body mass index, *HCs* healthy controls

*Statistical significance between the three groups at *P* value < 0.05

^a^Ampersand symbol (&) indicates significant difference between HCs and the ALS-CI subgroup; hash symbol (#) indicates a significant difference between the ALS-CN and ALS-CI subgroups

### HRV differences between ALS-CN and ALS-CI

The mean [max–min] HR (76 [48–114] vs. 70 [58–91] bpm; *P* = 0.032) was higher in the ALS-CI subgroup than in the HC group. In addition, the means for the following parameters were lower in the ALS-CI subgroup than in the HCs group: RRI (776.1 [669.6–1157.4] vs. 853.4 [646.4–1032.9] ms; *P* = 0.017), SDNN (115 [65–226] vs. 131.5 [84–175] ms; *P* = 0.013), SDNN Index (47 [19–94] vs. 51.5 [38–73] ms; *P* = 0.044), and VLF power (1184.3 [245.9–7620.2] vs. 1856.1 [970.6–4485.3] ms^2^; *P* = 0.012). Mean [max–min] TP (1814.7 [354.3–8895.5] ms^2^) was lower in the ALS-CI subgroup than in both the HC group (2363.2 [1444.7–5828.6] ms^2^; *P* = 0.036) and ALS-CN subgroup (2402.0 [621.6–5735.7] ms^2^; *P* = 0.048). Although a significant difference in LF power existed among the three groups (*P* = 0.035), no significant inter-group differences were found (Table 2; post-hoc comparisons between each two groups are shown in ESM Table S2). After controlling for age, gender, and education with ANCOVA, the SDNN Index (*P* = 0.020), TP (*P* = 0.001), VLF power (*P* = 0.002), LF power (*P* < 0.001), and HF power (*P* = 0.008) were different between the three groups (Table 2). Similar group differences were also identified when ECG data were separately analyzed for the daytime and nighttime periods; details are presented in ESM Table S5.

**Table 2:** Comparison of heart rate and heart rate variability parameters between patients with amyotrophic lateral sclerosis and healthy controls

Heart rate and heart rate variability parameters	Study (sub)groups			*P* value^b^	*P*’ value^c^
	HCs (*n* = 30)	ALS-CN (*n* = 28)	ALS-CI (*n* = 37)^a^		
Mean HR (bpm)	70 [58–91]	71.5 [61–93]	76 [48–114]^&^	0.035*	0.602
RRI (ms)	853.4 [646.4–1032.9]	813.3 [626.8–962.7]	776.1 [669.6–1157.4]^&^	0.022*	0.947
*24-h (time domain)*					
SDNN (ms)	131.5 [84–175]	130 [82–188]	115 [65–226]^&^	0.013*	0.069
SDANN (ms)	119.5 [71–164]	110 [67–166]	99 [55–174]	0.050	0.096
SDNN Index (ms)	51.5 [38–73]	51.5 [26–80]	47 [19–94]^&^	0.025*	0.020*
RMSSD (ms)	30 [17–88]	27 [19–89]	29 [11–97]	0.814	0.747
pNN50 (%)	5.5 [0–25]	3.5 [1–24]	3 [0–49]	0.369	0.220
*24-h (frequency domain)*					
TP (ms^2^)	2363.2 [1444.7–5828.6]	2402.0 [621.6–5735.7]	1814.7 [354.3–8895.5]^&,#^	0.015*	0.001*
VLF power (ms^2^)	1856.1 [970.6–4485.3]	1616.1 [440.0–4091.0]	1184.3 [245.9–7620.2]^&^	0.012*	0.002*
LF power (ms^2^)	374.6 [187.9–1070.8]	430.75 [110.7–1245.9]	299.6 [77.0–1018.4]	0.035*	< 0.001*
HF power (ms^2^)	159.7 [50.2–447.0]	136.7 [45.6–1103.7]	110.5 [13.6–526.2]	0.126	0.008*
LFnu	69.1 [49.0–82.4]	71.1 [12.7–87.2]	71 [35.5–88.2]	0.865	0.248
HFnu	28.3 [13.6–46.1]	26.2 [12.0–53.9]	25.3 [10.4–48.8]	0.683	0.783
LF/HF	2.4 [1.1–6.1]	2.8 [0.6–7.2]	2.8 [0.7–8.5]	0.643	0.516

* ALS* Amyotrophic lateral sclerosis, *ALS-CI* ALS patients with impaired cognition, *ALS-CN* ALS patients with normal cognition, *HCs* healthy controls, *HF power* high-frequency power, *LF/HF* ratio of LF power to HF power, *LFnu* LF power in normalized units, *HRV* heart rate variability, *LF power* low-frequency power, *pNN50* percentage difference between adjacent NN intervals > 50 ms, *RMSSD* square root of mean sum-of-squares of differences between adjacent NN intervals, *RRI* RR-interval, *SDANN* standard deviation of average NN intervals in all 5-min segments, *SDNN* standard deviation of all normal-to-normal (NN) intervals, *SDNN Index* mean standard deviations of all NN intervals for each 5-min segment in a 24-h HRV recording, *TP* total power, *VLF power* very low-frequency power

^a^Ampersand symbol (&) indicates significant difference between the HC group and the ALS-CI subgroup; the hash symbol (#) indicates a significant difference between the ALS-CN and ALS-CI subgroups

^b^Statistical significance between the three groups at *P* value < 0.05 according to Kruskal–Wallis test

^c^Statistical significance between the three groups at *P* value < 0.05 after Blom transformation ANCOVA was used to control for age, gender, and education level

Statistical significance between the three groups at P value < 0.05

### Correlations between cognitive domain scores and HRV parameters in ALS-CN patients

After adjusting for age, gender, and educational level, language ability in patients with ALS-CN showed a negative correlation with mean HR (*rs* = − 0.621; *P* = 0.001), and positive correlations with RRI (*rs* = 0.570; *P* = 0.003), SDNN (*rs* = 0.601; *P* = 0.001), SDANN (*rs* = 0.543; *P* = 0.005), TP (*rs* = 0.533; *P* = 0.006), VLF power (*rs* = 0.497; *P* = 0.011), and LF power (*rs* = 0.444; *P* = 0.026; Table 3). Visuospatial function in ALS-CN patients positively correlated with the SDNN Index (*rs* = 0.411, *P* = 0.041; Table 3). No associations were found between total ECAS or the other three ECAS cognitive domains and any of the 14 HRV/HR measures in this group of ALS patients.

**Table 3:** Partial correlations between heart rate and heart rate variablility parameters and Edinburgh Cognitive and Behavioral ALS Screen total and cognitive domain scores in patients with amyotrophic lateral sclerosis with normal cognition

Heart rate and heart rate variablility parameters	Total ECAS score		Language domain score		Fluency domain score		Executive function domain score		Memory domain score		Visuospatial function domain score	
	*r*	*P* value	*r*	*P* Value	*r*	*P* value	*r*	*P* value	*r*	*P* value	*r*	*P* Value
Mean HR	− 0.249	0.231	− 0.621	0.001*	0.153	0.466	− 0.213	0.307	0.091	0.664	− 0.390	0.054
RRI (ms)	0.219	0.293	0.570	0.003*	− 0.154	0.461	0.190	0.364	− 0.083	0.694	0.330	0.107
*24-h (time domain)*												
SDNN (ms)	0.270	0.193	0.601	0.001*	− 0.075	0.720	0.093	0.659	0.127	0.544	0.363	0.075
SDANN (ms)	0.250	0.228	0.543	0.005*	− 0.078	0.710	0.111	0.597	0.102	0.629	0.287	0.164
SDNN Index (ms)	− 0.043	0.838	0.287	0.165	− 0.039	0.854	− 0.224	0.281	0.020	0.923	0.411	0.041*
RMSSD (ms)	− 0.140	0.504	− 0.171	0.415	0.221	0.288	− 0.279	0.177	- 0.006	0.978	0.216	0.300
pNN50 (%)	0.036	0.864	0.154	0.461	0.128	0.541	− 0.122	0.563	0.040	0.848	0.196	0.348
*24-h (frequency domain)*												
TP (ms^2^)	0.102	0.627	0.533	0.006*	− 0.236	0.256	− 0.024	0.911	0.075	0.721	0.328	0.109
VLF power (ms^2^)	− 0.014	0.945	0.497	0.011*	− 0.303	0.141	− 0.135	0.521	0.050	0.814	0.232	0.265
LF power (ms^2^)	0.072	0.732	0.444	0.026*	− 0.219	0.293	− 0.023	0.915	0.044	0.835	0.332	0.105
HF power (ms^2^)	0.063	0.764	0.118	0.573	0.110	0.602	0.008	0.969	− 0.075	0.721	0.262	0.205
LFnu	0.011	0.959	0.042	0.843	− 0.183	0.380	0.025	0.906	0.118	0.574	0.080	0.706
HFnu	0.106	0.614	0.078	0.712	0.303	0.141	0.038	0.858	− 0.132	0.529	0.064	0.763
LF/HF	− 0.046	0.828	− 0.033	0.874	− 0.177	0.397	− 0.029	0.892	0.112	0.594	0.055	0.794

* ALS* Amyotrophic lateral sclerosis,* ECAS* Edinburgh Cognitive and Behavioral ALS Screen, *HF power* high-frequency power, *LF/HF* ratio of LF power to HF power, *LFnu* LF power in normalized units, *LF power* low-frequency power, *pNN50* percentage difference between adjacent NN intervals > 50 ms,* r* partial correlation coefficient, *RMSSD* square root of mean sum-of-squares of differences between adjacent NN intervals, *RRI* RR-interval, *SDANN* standard deviation of average NN intervals in all 5-min segments, *SDNN* standard deviation of all normal-to-normal (NN) intervals, *SDNN Index* mean standard deviations of all NN intervals for each 5-min segment in a 24-h HRV recording, *TP* total power, *VLF power* very low-frequency power

*Statistical significance at *P* value < 0.05. Adjustments were made for age, gender, and educational level

### Correlation between cognitive domain scores and HRV parameters in ALS-CI patients

After adjusting for age, gender, and educational level, we found that out of all the HRV measures, the ECAS total score (*rs* = − 0.443; *P* = 0.009) and the ECAS executive function domain score (*rs* = − 0.412; *P* = 0.015) in ALS-CI patients were negatively associated only with RMSSD and the visuospatial function was positively correlated with LFnu (*rs* = 0.340; *P* = 0.049), as shown in Table 4. No associations were observed between the scores of the other three ECAS cognitive domains and any of the 14 HRV/HR measures.

**Table 4:** Partial correlations between heart rate and heart rate variablility parameters and Edinburgh Cognitive and Behavioral ALS Screen total and cognitive domain scores in patients with amyotrophic lateral sclerosis with impaired cognition

Heart rate and heart rate variablility parameters	Total ECAS score		Language domain score		Fluency domain score		Executive function domain score		Memory domain score		Visuospatial function domain score	
	*r*	*P* value	*r*	*P* value	*r*	*P* value	*r*	*P* value	*r*	*P* value	*r*	*P* value
Mean HR	− 0.044	0.803	0.050	0.778	− 0.243	0.166	0.024	0.893	0.101	0.571	0.102	0.565
RRI (ms)	− 0.059	0.742	0.026	0.884	0.163	0.356	− 0.094	0.595	− 0.228	0.194	− 0.114	0.519
*24-h (time domain)*												
SDNN (ms)	− 0.159	0.370	0.024	0.895	0.032	0.856	− 0.222	0.207	− 0.097	0.586	− 0.200	0.256
SDANN (ms)	− 0.052	0.770	− 0.018	0.917	0.069	0.700	− 0.138	0.437	0.042	0.814	− 0.162	0.359
SDNN-Index (ms)	− 0.280	0.109	0.068	0.700	− 0.051	0.777	− 0.305	0.079	− 0.223	0.205	− 0.215	0.222
RMSSD (ms)	− 0.443	0.009*	− 0.006	0.971	− 0.250	0.154	− 0.412	0.015*	− 0.199	0.259	− 0.271	0.121
pNN50 (%)	− 0.252	0.150	0.061	0.733	− 0.128	0.472	− 0.109	0.538	− 0.289	0.098	− 0.231	0.190
*24-h (frequency domain)*												
TP (ms^2^)	− 0.090	0.613	0.069	0.699	0.012	0.947	− 0.073	0.682	− 0.161	0.363	− 0.063	0.722
VLF power (ms^2^)	− 0.036	0.839	0.121	0.494	− 0.007	0.970	− 0.005	0.978	− 0.145	0.412	− 0.034	0.847
LF power (ms^2^)	− 0.288	0.098	0.035	0.844	0.033	0.854	− 0.293	0.093	− 0.328	0.058	− 0.193	0.274
HF power (ms^2^)	− 0.252	0.151	− 0.047	0.793	0.026	0.882	− 0.173	0.329	− 0.274	0.117	− 0.281	0.107
LFnu	0.127	0.475	0.041	0.816	0.012	0.948	0.063	0.724	0.063	0.723	0.340	0.049*
HFnu	− 0.078	0.660	− 0.072	0.684	0.093	0.600	− 0.038	0.830	− 0.081	0.650	− 0.338	0.051
LF/HF	0.067	0.706	0.159	0.369	− 0.118	0.506	0.027	0.881	0.064	0.721	0.314	0.070

* ALS* Amyotrophic lateral sclerosis,* ECAS* Edinburgh Cognitive and Behavioral ALS Screen, *HF power* high-frequency power, *LF/HF* ratio of LF power to HF power, *LFnu* LF power in normalized units, *LF power* low-frequency power, *pNN50* percentage difference between adjacent NN intervals > 50 ms,* r* partial correlation coefficient, *RMSSD* square root of mean sum-of-squares of differences between adjacent NN intervals, *RRI* RR-interval, *SDANN* standard deviation of average NN intervals in all 5-min segments, *SDNN* standard deviation of all normal-to-normal (NN) intervals, *SDNN Index* mean standard deviations of all NN intervals for each 5-min segment in a 24-h HRV recording, *TP* total power, *VLF power* very low-frequency power

*Statistically significant difference at *P* value < 0.05. Adjustments were made for age, gender, and educational level

### Associations of demographic and HRV parameters with cognitive status

Multivariate logistic regression showed that lower education level (odds ration [OR] 0.699, 95% confidence interval [CI] 0.559–0.873; *P* = 0.002) and SDNN ≤ 100 ms (OR 0.112, 95% CI 0.014–0.894; *P* = 0.039) were associated with cognitive impairment while age (*P* = 0.588), gender (*P* = 0.342), disease duration (*P* = 0.537) and ALSFRS-R scores (*P* = 0.467) did not yield significant results (Table 5).

**Table 5:** Multivariate logistic regression examining the associations of demographic factors and the standard deviation of all normal-to-normal intervals with cognitive impairment in patients with amyotrophic lateral sclerosis

Parameters	Group	*β*	Odds ratio	95% confidence interval	*P* value
Age (year)	–	0.020	1.020	0.948–1.098	0.588
Gender	Male^a^				
	Female	0.632	1.881	0.511–6.922	0.342
Education level (year)		−0.358	0.699	0.559–0.873	0.002*
Disease duration	–	0.016	1.017	0.965–1.071	0.537
ALSFRS-R	–	0.043	1.044	0.929–1.173	0.467
SDNN (ms)	SDNN ≤ 100^a^				
	SDNN > 100	−2.186	0.112	[0.014–0.894]	0.039*

*ALSFRS-R* Amyotrophic Lateral Sclerosis Functional Rating Scale-Revised,* SDNN* standard deviation of all normal-to-normal intervals

*Statistically significant difference from reference group at *P* value < 0.05

^a^Reference group

### Associations of cognitive status and SDNN with survival in patients with ALS

Kaplan–Meier estimator showed baseline SDNN ≤ 100 ms was associated with poorer survival in ALS (*P* = 0.003; ESM Fig. 1a; ESM Table S3), while cognitive impairment had borderline association with poorer survival (*P* = 0.072) (ESM Table S3) in ALS. Cox regression model A, which included age, gender, education level, and SDNN, showed that SDNN ≤ 100 ms had a borderline association with poorer survival (*P* = 0.066; ESM Fig. 1b; Table 6). In contrast, Cox regression model B, which included age, gender, education level, and cognitive status, showed that cognitive impairment was associated with poorer survival (*P* = 0.010; Table S4). Older age was associated with poorer survival in Cox regression models A and B (*P* = 0.001, Table 6; *P* < 0.001, ESM Table S4).

**Table 6:** Associations of demographic factors and the standard deviation of all normal-to-normal intervals grouping with survival of patients with amyotrophic lateral sclerosis by multivariate Cox regression analysis model A

Parameters	Group	*β*	HR	95% CI	*P* Value
Age (year)	–	0.101	1.106	[1.040–1.176 ]	0.001*
Gender	Male^a^				
	Female	− 0.182	0.834	[0.311–2.237 ]	0.718
Education level (year)	–	− 0.020	0.980	[0.886–1.084 ]	0.700
SDNN (ms)	SDNN ≤ 100^a^				
	SDNN > 100	− 1.008	0.365	[0.125–1.069 ]	0.066

* SDNN* Standard deviation of all normal-to-normal intervals

*Statistically significant difference from reference group at *P* value < 0.05

^a^Reference group

## Discussion

Our study showed increased mean HR and a decrease in RRI and multiple HRV parameters among ALS patients with cognitive impairment (ALS-CI subgroup) compared to HCs. In contrast, no significant differences were found between ALS patients without cognitive impairment (ALS-CN subgroup) and the HCs. Consistent with previous research findings [6, 10, 12], the ALS patients with cognitive impairment in the present study exhibited lower education levels. Even after controlling for age (which was similar between the three study groups), gender, and education level (the ALS-CI group had fewer years of education than the HCs and ALS-CN group), multiple HRV parameters, including SDNN Index, TP, VLF power, and LF power, remained different between the three groups, suggesting cognitive impairment in ALS was related to changes in cardiac autonomic control independent of any possible influence of age, gender, and education. While a decrease in RRI may indicate a reduction in cardiovagal tone [30], reductions in SDNN, SDNN index, and TP indicate an overall decrease in autonomic cardiac modulations [30, 31]. Although VLF power can be influenced by various factors, such as the intrinsic cardiac nervous system, physical activity, thermoregulation, renin-angiotensin system, and endothelial system, as well as the autonomic nervous system, LF powers could reflect both sympathetic and parasympathetic modulations of the heart [30, 31]. Interestingly, HF power significantly differed between the groups after controlling for age, gender, and education, supporting a reduction in cardiovagal modulation in the ALS-CI group [30]. Overall, our findings support a generalized reduction in cardiac autonomic modulations with potentially reduced cardiovagal tone among ALS patients with cognitive impairment.

The associations between language performance and HRV parameters, such as mean HR and RRI, indicate that increased parasympathetic activity (or reduced sympathetic activity) correlated with better language abilities. Furthermore, higher cardiac autonomic modulation (reflected in SDNN, SDANN, TP, VLF power, and LF power) in the ALS-CN group was positively correlated with language performance [30]. The size of these correlations (e.g., absolute coefficient value ranging from 0.444 to 0.621) indicates moderate relationships, suggesting that autonomic modulation may play a significant role in supporting cognitive functions such as language. Additionally, visuospatial function showed positive associations with the SDNN Index and borderline associations with SDNN (positively) and mean HR (negatively), with overall coefficient size indicating weak to moderate relationships, suggesting in turn a link between cardiac autonomic modulation and visuospatial abilities. While there is limited data directly linking language to HRV, Frewen et al. [42] reported correlations between HRV parameters (e.g., mean HR, SDNN, LF powers, and LF/HF ratio) and language scores assessed via MOCA in older adults. These findings align with the moderate correlations observed in our study, emphasizing the relevance of autonomic function in language performance. In the present study we did not find significant correlations between HRV parameters and executive function in ALS patients who had normal cognition. However, previous findings from healthy populations have highlighted autonomic-cognitive correlations, particularly in executive functions evaluated by alternative cognitive tests [14, 43]. This discrepancy might be due to sample size limitations or differences in the cognitive questionnaires used.

In contrast, the ALS-CI cohort showed distinct patterns. Notably, associations between language performance and HRV parameters were absent in this group of patients, highlighting a potential decoupling of autonomic-cognitive relationships in the presence of cognitive impairment. For visuospatial function, positive associations were observed with LFnu (sympathetic modulation) and borderline associations were observed with HFnu (parasympathetic modulation) and LF/HF ratio (sympatho-vagal balance), with correlation coefficients > 0.3 suggesting a weak relationship. Conversely, reductions in parasympathetic modulation (e.g., RMSSD) were positively associated with executive function and general cognition, with correlation coefficients > 0.4 suggesting a moderate relationship. This pattern, albeit counterintuitive compared to findings in our cognitively intact ALS patients, and the commonly observed pattern of increased sympathetic and decreased parasympathetic activity relating to poorer cognitive performance in other populations without dementia, TBI, and stroke [14], suggests a shift in autonomic-cognitive interactions in ALS patients with cognitive impairment. Of note, the Framingham Offspring cohort demonstrated that higher SDNN and RMSSD were associated with reduced dementia risk among older individuals during follow-up [44]. Although previous studies have not investigated the correlations between HRV parameters and cognitive function in ALS patients, several studies have revealed associations between HRV parameters and cognitive function in populations with diverse pathologies [15, 16, 18]. Specifically, impaired executive function has been linked to reduced parasympathetic modulation in patients with chronic fatigue syndrome [18]. Higher SDNN, RMSSD, LF power, and HF power in patients with TBI have been associated with better social cognition [16]. On the contrary, the sympathovagal imbalance was associated with decreased visuospatial and executive performance [15].

The scarcity of significant correlations in the ALS-CI group, compared to ALS-CN patients, could partially be attributed to our limited sample size. However, it is also plausible that the typical autonomic-cognitive associations observed in our ALS-CN group may alter with disease progression, reflecting changes in neural networks and autonomic regulation mechanisms in ALS patients who have developed cognitive impairment. This possibility is further corroborated by our finding that ALS patients with SDNN ≤ 100 ms already had an increased risk of cognitive impairment at baseline. We postulate that a reduction in parasympathetic modulation and shifting toward relative sympathetic dominance could be compensatory changes for cognitive impairment.

Our results showed that in addition to age [45], cognitive impairment was related to shorter survival in ALS patients, based on multi-variable Cox regression analysis. William et al. [27] found that dementia was linked to shorter survival primarily in ALS patients with disinhibited or apathetic behaviors. Fan et al. [26] found that ALS patients with a behavioral variant of FTD had a worse prognosis than those with pure motor or cognitive and behavioral symptoms. In contrast, the prognosis between the latter two groups did not differ. Noteworthy, ALS patients are also at risk of experiencing cardiovascular events and sudden death [46, 47]. We found baseline SDNN ≤ 100 ms was associated with a shorter survival in ALS patients (ESM Fig. 1a); however, these associations became borderline after including age, gender, and education in the Cox regression model (Table 6; ESM Fig. 1b). This result could be explained by the strong correlation between SDNN and age [30], as gender or education level was not associated with survival in any of the models. Previous studies have shown the associations between abnormal HRV and worse clinical outcomes in different pathologies [28–32, 38]. One recent study investigating the associations of autonomic symptoms on survival in ALS patients found that urinary complaints were independent factors of poor survival. However, this study did not evaluate baseline HRV parameters for the efficacy of prognosis [25]. Kleiger et al. found patients after acute myocardial infarction had the highest mortality with SDNN ≤ 50 ms compared to those with SDNN > 100 ms [38, 40, 41]. Our results support the premise that even suboptimal HRV (none of our patients had SDNN ≤ 50 ms) could be associated with poorer survival in ALS patients and suggest the potential value of testing HRV in ALS patients. More extensive studies are needed to confirm our findings.

Previous imaging studies have provided evidence on the substrates underlying cognitive impairment in ALS patients [48–50]. One study investigating motor neuron disease revealed that in comparison to ALS patients with pure motor symptoms and HCs, ALS patients with additional cognitive and behavioral symptoms experienced cortical thinning in the cortex of the cingulate gyrus, orbitofrontal lobe, and temporal lobe, as well as a loss of white matter in the corpus callosum and upper longitudinal tract [50]. Using imaging cluster analysis, the authors of another study classified ALS into three distinct clusters: the pure motor cluster characterized by exclusively motor involvement; the frontotemporal (FT) cluster involving the orbitofrontal and temporal lobes; and the cingulate-parietal-temporal (CPT) cluster involving the posterior cingulate cortex, parietal white matter, temporal operculum, and cerebellum. The FT and CPT clusters were associated with more significant cognitive impairment than the pure motor clusters, including a higher frequency of language and executive dysfunction. In contrast, memory dysfunction was more prominent within the FT cluster [48]. In summary, cognitive function in ALS is regulated by the frontal lobe, cingulate gyrus, corpus callosum, and temporal lobe.

The central autonomic network (CAN), which encompasses the brainstem and forebrain regions, regulates the baseline function of the autonomic nervous system as well as its response to environmental changes [51]. Critical components of CAN, including the cingulate gyrus, prefrontal cortex, hypothalamus, insula, hippocampus, and brainstem structures, are involved in modulating cardiac autonomic function [52]. Therefore, there is a significant overlap between the brain regions regulating cognition in ALS patients [49] and those comprising the CAN [52]. This overlap is likely supported by the positive associations between language and multiple HRV parameters in ALS patients without cognitive impairment and the reduced overall cardiac autonomic modulation and altered cognitive and autonomic association patterns found in ALS patients with cognitive impairment. Our findings suggest that even in ALS patients who do not meet the diagnostic criteria for FTD [4], cognitive and autonomic structures may have already been involved in the disease process, leading to an altered orchestration of cognitive and cardiac autonomic control [53] and possibly a poorer prognosis.

Our study has several limitations. Firstly, the sample size was relatively small, which limited the number of variables in the survival analyses and which might have contributed to the borderline* P* value of a few analyses; however, rigorous inclusion and exclusion criteria were implemented to mitigate confounding factors that could impact cardiac autonomic function. Also, the limited number of significant correlations between HRV parameters and ECAS domain scores among two ALS subgroups might not have reflected the complete profile of changes in autonomic cognitive associations in ALS patients with cognitive impairment due to the small sample size. Moreover, the median ALSFRS-R scores for both ALS subgroups were relatively high (42 for ALS-CN and 41 for ALS-CI); thus, we could not fully evaluate the patient population with a much lower ALSFRS-R score. Additionally, we did not prospectively monitor cardiac autonomic changes during follow-up. Moreover, the lack of screening for potential ALS-related genetic mutations limits the generalization of our findings. However, it is worth noting that previous studies found a relatively low frequency of C9ORF72, TARDBP, and FUS mutations in the Chinese population [54, 55].

## Conclusion

In the present study, ALS patients with clinical cognitive impairment (ALS-CI group) exhibited reduced RRI and HRV parameters compared to healthy people (HCs); they also likely had altered cognitive-autonomic coupling compared to the ALS patients with intact cognition (ALS-CN group). SDNN ≤ 100 ms affected cognitive impairment in ALS patients as early as at baseline, while SDNN ≤ 100 ms was borderline-wise, and cognitive impairment was associated with poorer survival in ALS patients during a median 2-year follow-up, highlighting the clinical value of monitoring cardiac autonomic function and cognitive status in ALS patients.

## Supplementary Information

Below is the link to the electronic supplementary material.

Supplementary file1 (DOCX 16 KB)

Supplementary file2 (TIF 27881 KB)

Supplementary file3 (DOCX 33 KB)

## Data Availability

The datasets generated and analyzed during the current study are available upon reasonable request from the corresponding author.
