# Supplementary material for: Association between cardiac autonomic dysfunction, cognitive impairment, and survival in patients with amyotrophic lateral sclerosis
Source: Clin Auton Res. 2025 Mar 8;35(3):465–76. doi: 10.1007/s10286-025-01112-0 (PMC12137521; doi:10.1007/s10286-025-01112-0)
Supplement: Supplementary file 1 — Supplementary file1 (DOCX 16 KB) [file 10286_2025_1112_MOESM1_ESM.docx]

**Fig 1** a) Survival curve of ALS patients based on SDNN grouping via Kaplan-Meier estimator b) Forest graph for the survival of ALS patients by multivariable Cox regression analysis.
