# Supplementary figures and images for: Association between cardiac autonomic dysfunction, cognitive impairment, and survival in patients with amyotrophic lateral sclerosis
Source: Clin Auton Res. 2025 Mar 8;35(3):465–76. doi: 10.1007/s10286-025-01112-0 (PMC12137521; doi:10.1007/s10286-025-01112-0)

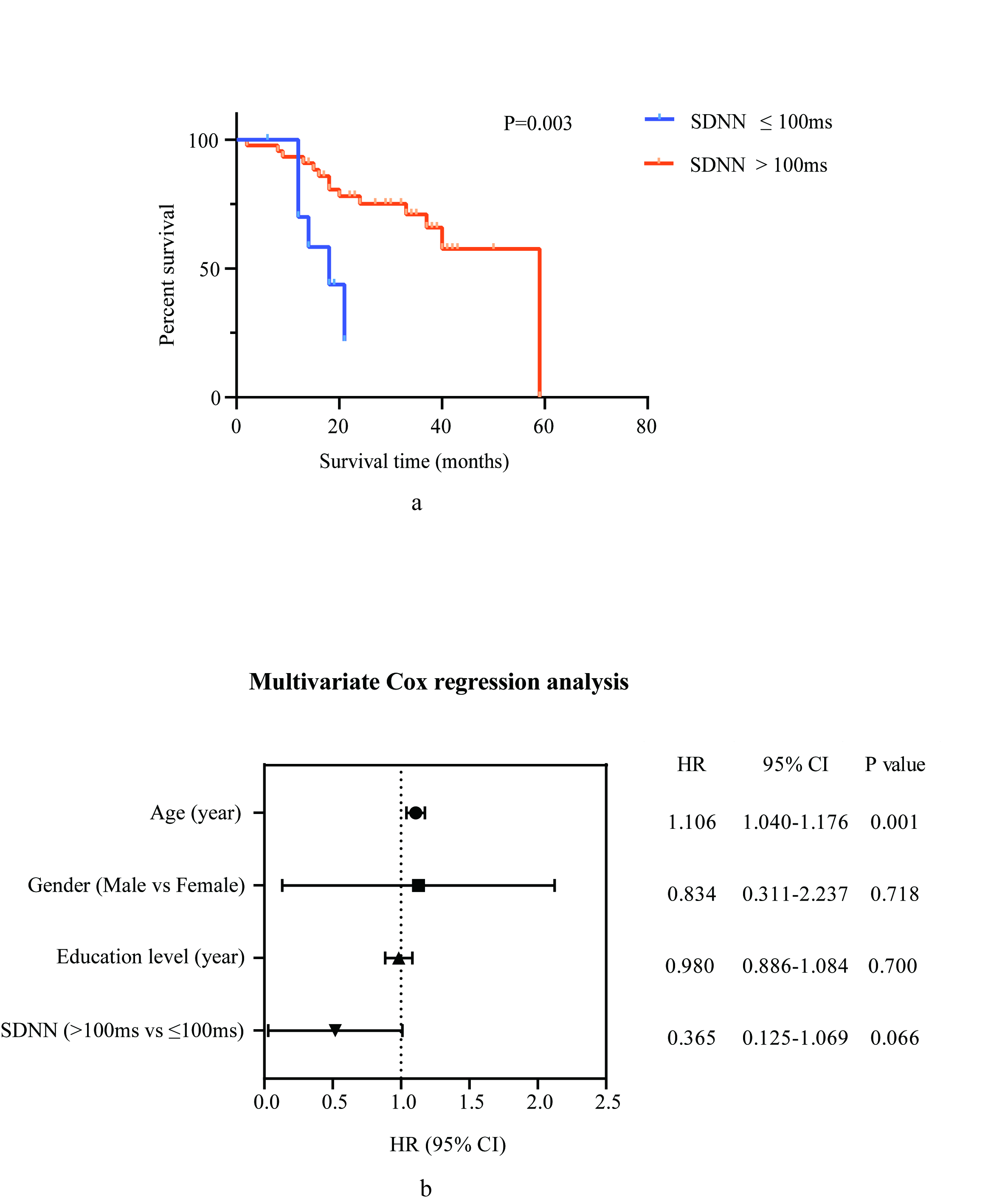

Supplement: Supplementary file 2 — Supplementary file2 (TIF 27881 KB) [file 10286_2025_1112_MOESM2_ESM.tif]
