# Supplementary material for: Association between cardiac autonomic dysfunction, cognitive impairment, and survival in patients with amyotrophic lateral sclerosis
Source: Clin Auton Res. 2025 Mar 8;35(3):465–76. doi: 10.1007/s10286-025-01112-0 (PMC12137521; doi:10.1007/s10286-025-01112-0)
Supplement: Supplementary file 3 — Supplementary file3 (DOCX 33 KB) [file 10286_2025_1112_MOESM3_ESM.docx]

**Table S1**. Heart rate variability parameters of time and frequency domains

| Parameters | Description | Implications |
| --- | --- | --- |
| RRI | RR-interval | the reciprocal of heart rate |
| **Time Domain** |  |  |
| SDNN (ms) | Standard deviation of all normal-to-normal (NN) intervals | Overall autonomic function |
| SDANN (ms) | Standard deviation of the averages of NN intervals in all 5-min segments | Overall autonomic function |
| SDNN-Index (ms) | Mean of the standard deviations of all the NN intervals for each 5 min segment of a 24 h HRV recording | Overall autonomic function |
| RMSSD (ms) | Square root of the mean of the sum of squares of differences between adjacent NN intervals | Parasympathetic activity |
| pNN50 (%) | Percentage difference between adjacent NN intervals that are greater than 50 ms | Parasympathetic activity |
| **Frequency Domain** |  |  |
| TP (ms^2^) | Total-Power (0-0.40Hz) |  |
| VLF-Power (ms^2^) | Very Low Frequency Power (0.003-0.04Hz) | intrinsic cardiac nervous system; sympathetic and parasympathetic activity; influences from physical activity, thermoregulation, renin-angiotensin system, and endothelial system |
| LF-Power (ms^2^) | Low-Frequency power (0.04-0.15Hz) | Both sympathetic and parasympathetic activity |
| HF-Power (ms^2^) | High-Frequency power (0.15-0.40Hz) | Parasympathetic activity |
| LF-Power in normalized units, LFnu | LF Power/(LF Power + HF Power)*100.0 | Both sympathetic and parasympathetic activity |
| HF-Power in normalized units, HFnu | HF Power/(LF Power + HF Power) *100.0 | Parasympathetic activity |
| LF/HF | Ratio of LF power to HF power | Sympatho-parasympathetic balance |

**Table S2**. Post-hoc comparison results for ALS-CI, ALS-CN, and HC

|  | *P1* | *P2* | *P3* |
| --- | --- | --- | --- |
| Education (year) | <0.001* | 0.001* | >0.999 |
| **HRV parameters** |  |  |  |
| Mean-HR (bpm) | 0.032* | 0.438 | 0.944 |
| RRI (ms) | 0.017* | 0.530 | 0.590 |
| **24-hour (time-domain)** |  |  |  |
| SDNN (ms) | 0.013* | 0.152 | >0.999 |
| SDNN-Index (ms) | 0.044* | 0.092 | >0.999 |
| **24-hour (frequency-domain)** |  |  |  |
| TP (ms^2^) | 0.036* | 0.048* | >0.999 |
| VLF-Power (ms^2^) | 0.012* | 0.156 | >0.999 |
| LF-Power (ms^2^) | 0.135 | 0.056 | >0.999 |

Abbreviations: HC, health controls; ALS-CN, ALS patients with normal cognition; ALS-CI, ALS patients with impaired cognition; HR, Heart rate; RRI, RR-interval; SDNN, Standard deviation of all normal-to-normal (NN) intervals; SDNN-Index, Mean standard deviations of all NN intervals for each 5-minute segment in a 24-hour HRV recording; TP, Total-Power; VLF-Power, Very-Low-Frequency Power; LF-Power, Low-Frequency power.

P Value: P1, ALS-CI vs HC; P2, ALS-CI vs ALS-CN; P3, ALS-CN vs HC

*Statistical significance at *P* value < 0.05.

**Table S3.** Associations of SDNN and cognitive status with survival of ALS patients by Kaplan-Meier estimator

| Parameters | Group | Mean survival time | 95% CI | Chi-square of Log-rank | P Value |
| --- | --- | --- | --- | --- | --- |
| SDNN (ms) | SDNN>100^&^ | 44.239 | [37.438-51.040] | 8.873 | 0.003* |
|  | SDNN≤100 | 17.046 | [14.265-19.827] |  |  |
| Cognitive status | ALS-CN^&^ | 47.660 | [38.558-56.762] | 3.231 | 0.072 |
|  | ALS-CI | 29.714 |  |  |  |

Abbreviations: ALS-CN, ALS patients with normal cognition; ALS-CI, ALS patients with impaired cognition, SDNN, standard deviation of all normal-to-normal (NN) intervals.

^&^reference group.

*Statistical significance at *P* value < 0.05.

**Table S4**. Associations of demographic factors and cognitive status with survival of ALS patients by multivariate Cox regression analysis model B

| Parameters | Group | B | HR | 95% CI | *P* Value |
| --- | --- | --- | --- | --- | --- |
| Age (year) | - | 0.138 | 1.148 | [1.072-1.229] | <0.001* |
| Gender | Male^&^ |  |  |  |  |
|  | Female | -0.105 | 0.900 | [0.350-2.314] | 0.827 |
| Education level (year) | - | 0.063 | 1.065 | [0.947-1.197] | 0.295 |
| Cognitive status | ALS-CN^&^ |  |  |  |  |
|  | ALS-CI | 1.705 | 5.500 | [1.509-20.042] | 0.010* |

Abbreviations: ALS-CN, ALS patients with normal cognition; ALS-CI, ALS patients with impaired cognition.

^&^reference group.

*Statistical significance at *P* value < 0.05.

**Table S5**. Comparison of HRV parameters between ALS-CI, ALS-CN, and HC in daytime and nighttime

|  | HC  n=38 | ALS-CN  n=28 | ALS-CI  n=37 | *P* Value | *P’* Value |
| --- | --- | --- | --- | --- | --- |
| **Daytime** |  |  |  |  |  |
| **time-domain** |  |  |  |  |  |
| SDNN (ms) | 103.0  [67.0-149.0] | 102.0  [66.0-148.0] | 97.0  [57.0-236.0] | 0.044* | 0.169 |
| RMSSD (ms) | 28.0  [13.0-104.0] | 28.5  [16.0-68.0] | 27.0  [10.0-71.0] | 0.812 | 0.548 |
| pNN50 (%) | 5.0  [0-22.0] | 3.0  [0-20.0] | 2.0  [0-32.0] | 0.126 | 0.885 |
| **frequency-domain** |  |  |  |  |  |
| TP (ms^2^) | 2238.7  [863.7-4777.5] | 1978.6  [516.3-4841.2] | 1502.1  [394.9-6224.4]^&^ | 0.014* | 0.002* |
| VLF-Power (ms^2^) | 1635.6  [623.9-3586.3] | 1451.3  [356.2-3271.1] | 1140.9  [283.4-4679.3]^&^ | 0.009* | 0.005* |
| LF-Power (ms^2^) | 361.9  [146.0-966.0] | 383.4  [98.8-1240.5] | 292.4  [63.6-1084.3] | 0.036* | <0.001* |
| HF-Power (ms^2^) | 119.5  [48.4-318.5] | 123.6  [34.1-359.3] | 81.3  [10.3-413.3] | 0.132 | 0.004* |
| LFnu | 72.5  [50.4-83.1] | 73.1  [49.8-88.5] | 74.8  [30.0-89.6] | 0.743 | 0.199 |
| HFnu | 24.0  [12.0-40.9] | 23.4  [10.8-41.4] | 22.0  [9.2-47.4] | 0.709 | 0.683 |
| LF/HF | 3.0  [1.4-6.9] | 3.4  [1.2-8.2] | 3.6  [0.6-9.7] | 0.625 | 0.553 |
| **Night-time** |  |  |  |  |  |
| **time-domain** |  |  |  |  |  |
| SDNN (ms) | 88.5  [49.0-233.0] | 91.0  [55.0-137.0] | 84.0  [39.0-196.0] | 0.129 | 0.087 |
| RMSSD (ms) | 34.5  [16.0-79.0] | 32.5  [19.0-139.0] | 30.0  [10.0-135.0] | 0.235 | 0.380 |
| pNN50 (%) | 10.0  [0-33.0] | 8.5  [1.0-48.0] | 5.0  [0-46.0] | 0.152 | 0.052 |
| **frequency-domain** |  |  |  |  |  |
| TP (ms^2^) | 2815.8  [988.3-7845.6] | 2903.1  [814.2-9843.9] | 2106.0  [282.4-1777.6] | 0.043* | <0.001* |
| VLF-Power (ms^2^) | 1948.2  [653.4-6200.1] | 2231.8  [593.9-8404.5] | 1544.3  [179.7-15400.5] | 0.033* | 0.001* |
| LF-Power (ms^2^) | 463.3  [111.1-1281.1] | 495.3  [124.4-1416.0] | 361.0  [55.7-1301.3] | 0.057 | <0.001* |
| HF-Power (ms^2^) | 258.2  [53.2-827.4] | 229.0  [57.5-2517.0] | 178.9  [20.1-1105.7] | 0.097 | 0.010* |
| LFnu | 59.8  [35.2-83.6] | 66.0  [28.2-85.0] | 62.9  [26.6-89.2] | 0.276 | 0.807 |
| HFnu | 36.2  [14.5-59.6] | 30.6  [12.0-58.4] | 32.4  [9.4-58.8] | 0.120 | 0.757 |
| LF/HF | 1.6  [0.6-5.7] | 2.3  [0.5-6.2] | 2.0  [0.5-9.5] | 0.080 | 0.848 |

Abbreviations: HRV, heart rate variability; HC, health controls; ALS-CN, ALS patients with normal cognition; ALS-CI, ALS patients with impaired cognition; SDNN, Standard deviation of all normal-to-normal (NN) intervals; RMSSD, Square root of mean sum-of-squares of differences between adjacent NN intervals; pNN50, Percentage difference between adjacent NN intervals that are greater than 50 ms; TP, Total-Power; VLF-Power, Very-Low-Frequency Power; LF-Power, Low-Frequency power; HF-Power, High-Frequency power; LFnu, LF-Power in normalized units; HFnu, HF-Power in normalized units; LF/HF, Ratio of LF power to HF power.

Bold font indicates statistical significance between the three groups (p value<0.05). ^&^ significant difference between HC and ALS-CI.

*P* Value: Kruskal-Wallis test

*P’* Value: after Blom transformation ANCOVA was used to control age, gender, and education level

*Statistical significance between the three groups at *P* value < 0.05.

**Table S6**. Comparison of ECAS domain between ALS-CI and ALS-CN

|  | ALS-CN  n=28 | ALS-CI  n=37 | *P* Value |
| --- | --- | --- | --- |
| Total score | 93[82-136] | 63[24-81] | <0.001* |
| Language | 22[11-28] | 12[5-23] | <0.001* |
| Fluency | 20[8-24] | 12[0-24] | <0.001* |
| Executive function | 31[18-48] | 20[1-38] | <0.001* |
| Memory | 13[0-24] | 4[0-15] | <0.001* |
| Visuospatial function | 12[10-12] | 11[4-12] | 0.001* |

Abbreviations: ALS-CN, ALS patients with normal cognition; ALS-CI, ALS patients with impaired cognition; ECAS, Edinburgh Cognitive and Behavioral Screen.

*Statistical significance at *P* value < 0.05.
